# Leaf spectroscopy as a tool for predicting the presence of isoprene emissions and terpene storage in central Amazon forest trees

**DOI:** 10.1186/s13007-025-01400-w

**Published:** 2025-06-04

**Authors:** Michelle Robin, Flavia Machado Durgante, Caroline Lorenci Mallmann, Hilana Louise Hadlich, Christine Römermann, Lucas de Souza Falcão, Caroline Dutra Lacerda, Sérgio Duvoisin Jr., Florian Wittmann, Maria Teresa Fernandez Piedade, Jochen Schöngart, Eliane Gomes Alves

**Affiliations:** 1 Biogeochemical Processes Department, Max Planck Institute for Biogeochemistry, Jena, Germany; 2 Department of Wetlands Ecology, Karlsruhe Institute of Technology, Karlsruhe, Germany; 3 Department of Geosciences, Federal University of Santa Maria, Santa Maria, Brazil; 4 Department of Botany, National Institute of Amazonian Research, Manaus, Brazil; 5 Institute for Ecology and Evolution, Friedrich-Schiller University, Jena, Germany; 6 German Centre for Integrative Biodiversity Research (iDiv) Halle-Jena-Leipzig, Leipzig, Germany; 7 Senckenberg Institute for Plant Form and Function (SIP), Jena, Germany; 8 Department of Chemistry, University of Amazonas State, Manaus, Brazil; 9 Department of Climate and Environment, National Institute of Amazonian Research, Manaus, Brazil

**Keywords:** Spectroscopy, Volatile isoprenoids, Monoterpenes, Sesquiterpenes, BVOCs

## Abstract

**Background:**

Volatile isoprenoids (VIs), such as isoprene, monoterpenes, and sesquiterpenes, participate in various forest-atmosphere processes ranging from plant cell regulation to atmospheric particle formation. The Amazon Forest is the greatest and most diverse source of VI emissions, but the lack of leaf-level studies and the logistical challenges of measuring in such remote and highly biodiverse sites bring high levels of uncertainty to modeled emission estimates. Studies indicate that leaf spectroscopy is an effective tool for estimating leaf morphological, physiological, and chemical traits, being a promising tool for more easily assessing VI emissions from vegetation. In this study, we tested the ability of leaf reflectance spectroscopy to predict the presence of VI emissions and storage in central Amazon Forest trees. We measured leaf-level isoprene emission capacity (*E*_c_; emission measured at standard conditions: light of 1000 µmol m^− 2^ s^− 1^ photosynthetically active radiation and leaf temperature of 30 ˚C), stored monoterpene and sesquiterpene contents, and hyperspectral visible to short-wave infrared (VSWIR) reflectance from dry and fresh leaves of 175 trees from 124 species of angiosperms.

**Results:**

We found that dry leaf hyperspectral reflectance data, and fresh leaf reflectance measured at selected wavelengths (616, 694, and 1155 nm), predicted the presence of isoprene emissions with accuracies of 0.67 and 0.72, respectively. Meanwhile, fresh leaf hyperspectral reflectance data predicted monoterpene and sesquiterpene storage with accuracies of 0.65 and 0.67, respectively.

**Conclusions:**

Our results indicate the possibility of using spectral readings from botanical collections or field inventories to orient sampling efforts toward potential isoprene-emitting or terpene-storing trees, or to identify key spectral features (most informative selected wavelengths) for potential future incorporation into remote sensing models. The use of spectral tools for detecting potential isoprene-emitting and terpene-storing species can help to improve current VI emission datasets, reduce modeling emission uncertainties, and contribute to a better understanding of the roles of VIs within forest-atmosphere interactions, atmospheric chemistry, and the carbon cycle.

**Supplementary Information:**

The online version contains supplementary material available at 10.1186/s13007-025-01400-w.

## Background

Volatile isoprenoids (VIs; isoprene, monoterpenes, and sesquiterpenes) are central to a variety of processes across multiple scales, from plant cell regulation to secondary particle formation in the atmosphere. Isoprene (C_5_H_8_) emissions are known to be associated with increased thermotolerance [1–5], and studies currently suggest that this compound mediates both growth and defense responses under stress conditions [6] via coordination of gene expression and changes in transcription factors [7–11]. Monoterpenes (C_10_H_16_) and sesquiterpenes (C_15_H_24_) have diverse chemical signaling roles in herbivore defense, plant-plant communication, and attraction of pollinators [12–15], and recent studies have shown that isoprene also participates in herbivore defense [11, 16]. On top of that, VI emissions dominate global Biogenic Volatile Organic Compound (BVOC) fluxes [17, 18], with around 500 Tg C yr^− 1^ globally emitted by plants as isoprene [19]. These emissions impact atmospheric oxidative capacity - as these compounds are rapidly oxidized in the presence of ozone (O_3_), hydroxyl radical (OH), and nitrogen oxides (NOx) - and influence light scattering and precipitation through the formation of secondary organic aerosols [20–28].

Isoprene and monoterpenes are both produced in the chloroplastic methyl-erythritol 4-phosphate (MEP) pathway [29], and sesquiterpenes are produced in the cytosolic mevalonic acid (MVA) pathway [30]. Isoprene - and some light-dependent monoterpenes [31, 32] - are primarily produced and immediately emitted directly from photosynthetic carbon [33–36]. Meanwhile, sesquiterpenes and most monoterpenes form storage pools within the cell wall or inside specialized storage structures (e.g., resin ducts, oil glands, glandular trichomes), being slowly released under constitutive conditions or rapidly emitted upon breakage of these structures, e.g., under herbivore feeding [37–40]. The vast plant biomass and remarkable species diversity of the Amazon Forest [41, 42] make it the most abundant and chemically diverse source of VIs to the atmosphere [18, 27, 43]. However, this diversity contributes to significant uncertainty in modeled emission estimates [18, 27], which mostly rely on very simplified schemes of plant functional type (PFT) distributions (e.g., CLM4 model [44]), with a single emission factor value for each PFT. This, combined with the logistical challenges of obtaining in-situ measurements in the remote and often inaccessible regions of the forest, emphasizes the need for advancements in tools to facilitate the assessment and estimation of VI emissions.

Large-scale atmospheric isoprene fluxes have been indirectly estimated via inverse modeling from satellite measurements of formaldehyde (HCHO), a major intermediate product in isoprene oxidation [45–48]. Still, the assumptions about the oxidation chemistry linking isoprene to formaldehyde [48] and the influence of biomass burning- and non-isoprene BVOC-derived formaldehyde introduce much uncertainty to this method [49]. More recently, direct atmospheric observations of isoprene were made possible using the satellite-borne Cross-track Infrared Sounder, which showed improved sensitivity and higher resolution [49–51]. However, this method also faces challenges since it relies on estimates from models like MEGAN v2.1 [18] for initialization - from which errors or biases may propagate into the retrievals - and is subject to interference from other atmospheric components and meteorological factors [50]. Moreover, like formaldehyde retrieval, this method is not suitable for estimating more reactive VIs such as monoterpenes and sesquiterpenes [47].

Meanwhile, many studies indicate that leaf spectroscopy can be used to successfully identify taxonomic species [52–55] as well as predict leaf morphological, physiological, and chemical traits [56–68], perhaps being a promising tool for more easily assessing VI emissions from vegetation. The absorption of light by a leaf sample excites electrons within the leaf’s molecular constituents to a higher energy level, inducing vibrational transitions in molecular bonds (e.g., C–H, N–H, and C–O) at specific wavelengths in the visible (400–700 nm), near-infrared (700–1100 nm) and short-wave infrared (1100–2500 nm) regions of the electromagnetic spectrum [65, 69]. Leaf spectroscopy allows estimating various leaf structural and biochemical properties by examining these variations in light absorption and reflectance, and the unique spectral signatures they produce. This technique has yielded encouraging results in the estimation of VI emissions at leaf, canopy, and ecosystem levels [66, 70–72]. For instance, the photochemical reflectance index (PRI), calculated from leaf reflectance measured at 531 and 570 nm, revealed itself as a good indirect estimator of VI emissions at leaf [70] and canopy levels [71, 72]. However, this index is only useful for estimating light-dependent emissions from emitters, given that it is based on the observed relationship between emissions and light use efficiency (LUE) [70]. This method is based on the assumption that there is an enhanced supply of photosynthetic reducing power available for the production of light-dependent VIs under lower LUEs [73]. Hence, the PRI would not be useful to distinguish VI emitters from non-emitters or to predict the presence of stored VIs.

In that sense, we aimed to evaluate whether leaf reflectance spectroscopy could be used to predict the presence of VI emissions and storage in dry and fresh leaves from central Amazon Forest trees. We measured leaf-level isoprene emission capacity (*E*_c_; emission measured at standard conditions: light of 1000 µmol m^− 2^ s^− 1^ photosynthetically active radiation and leaf temperature of 30 ˚C), stored monoterpene and sesquiterpene contents, and hyperspectral visible to short-wave infrared reflectance (VSWIR) from dry and fresh leaves of 175 trees from 124 species of angiosperms. We tested the capacity of discriminant models built with raw and derived hyperspectral reflectance data and with reflectance measured at specific wavelengths (selected as most informative via stepwise feature selection analysis) from stacked leaves (hereafter referred to as “leaf reflectance”) to predict the presence or absence of isoprene *E*_c_ and mono-/sesquiterpene storage. We expected discriminant models to be able to predict the presence of mono-/sesquiterpene storage since these compounds are stored inside leaves and may display a spectral signature of their own. However, since isoprene is emitted immediately after its production and is not a storable compound, we expected discriminant models to be able to predict the presence of isoprene emissions due to its associations with other chemical and structural leaf traits [6, 74, 75]. The potential ability of dry and fresh leaf reflectance spectroscopy to predict the presence of VI emissions and storage may allow its use, for instance, as a pre-processing tool to assist the development of more efficient sampling designs based on spectral readings obtained from herbarium specimens (e.g., dry leaf reflectance measurements), or to orientate sampling efforts in the field (e.g., fresh leaf reflectance measurements) targeted at potential VI emitting or storing trees.

## Methods

### Study site

To test the capacity of discriminant models to predict the presence or absence of isoprene *E*_c_ and mono-/sesquiterpene storage, we performed measurements in an upland forest (locally called *terra firme*) permanent plot at the Amazon Tall Tower Observatory (ATTO) site in central Amazonia. The ATTO site is located about 150 km northeast of Manaus in the Uatumã Sustainable Development Reserve (USDR; 02° 08.9’ S, 59° 00.2’ W, 130 m a.s.l.). The site is situated in a humid tropical climate zone, with a mean annual temperature of 26.7 ºC and precipitation of 2376 mm, and is characterized by a pronounced wet season from December to May and a dry season from July to October, with a transitory moderately wet period in between the seasons [76]. Vegetation in the *terra firme* plot is dense (leaf area index of 5.3 m^2^ m^− 2^), mature, and non-flooded, with a mean canopy height of 35 m [43]. The soil is a highly weathered and well-drained ferralsol [77].

### Branch collection and sampling design

We obtained VSWIR reflectance measurements from leaves of branches sampled from 175 trees of 124 species of angiosperms. All trees occupied the upper canopy layer of the plot, were the most representative in terms of canopy dominance, and had been previously screened for the presence or absence of isoprene *E*_c_ and mono-/sesquiterpene storage [75]. The detailed number of trees where isoprene emissions or mono-/sesquiterpene storage was detected is presented in Table 1, and each tree measured constituted an individual observation. We sampled the trees and performed measurements between October 15 - November 9, 2022. This period corresponds to the transition between dry and wet seasons, when tree canopies are mostly composed of mature leaves [78], and variation in leaf age is expected to be low.

**Table 1:** Number of observations (*n*) of isoprene emitters and non-emitters, and mono-/sesquiterpene storing and non-storing trees used to build dry and fresh leaf reflectance-based models

		*n* total	*n* Non-emitter	*n* Emitter
***Isoprene emissions***	Dry	167	81	86
	Fresh	175	87	88
			*n* Non-storing	*n* Storing
***Monoterpene storage***	Dry	163	89	74
	Fresh	171	93	78
***Sesquiterpenes storage***	Dry	163	47	116
	Fresh	171	50	121

Given the logistical challenges of studying tall tropical trees, often exceeding 20 m in height, measurements were obtained from leaves of cut branches immediately placed in standard tap water (unfiltered, room temperature). This method provides a practical solution for conducting gas exchange and isoprene emission measurements, enabling the capture of key ecological processes without compromising leaf viability [75, 79–83]. For each tree, we collected one branch with a diameter of at least 2 cm from a sun-exposed area of the canopy to avoid shade-adapted leaves. Senescent, young, or visibly damaged leaves were excluded, ensuring that only physiologically active leaves were analyzed. After collection, the branch was immediately re-cut under water to prevent embolism, stored in a water bottle for transport, and re-cut once more under water at the field camp to restore xylem flow before isoprene *E*_c_ measurements.

The detection of isoprene emissions in a single leaf is sufficient to allow the classification of an individual tree as an isoprene emitter due to phylogenetic conservation of isoprene synthase encoding genes [84, 85]. Therefore, given time constraints, we selected one visibly mature and healthy leaf of the branch to measure leaf-level isoprene *E*_c_. We also selected four leaves to measure hyperspectral visible to short-wave infrared (VSWIR) reflectance, and between 10 and 20 leaves (fewer larger leaves and more smaller leaves were collected) that were immediately frozen in liquid nitrogen and further taken to Manaus for terpene storage analysis. Detailed descriptions of isoprene *E*_c_ and stored mono-/sesquiterpene measurements can be found in the supporting information (Methods S1, 2).

### Hyperspectral visible to short-wave infrared (VSWIR) reflectance measurements

Measurements of hyperspectral VSWIR reflectance were obtained with a field spectrometer (FieldSpec-4, Malvern Panalytical Ltd., formerly Analytical Spectral Devices, Boulder, USA). The instrument records 2151 reflectance values, at a spectral resolution of 3 nm from 350 to 700 nm, and 10 nm from 701 to 2500 nm, covering the visible, near-infrared, and short-wave infrared portions of the electromagnetic spectrum. Before each spectral reading, the instrument was calibrated for dark current and stray light, referenced to a calibration block (Spectralon; LabSphere, Durham, New Hampshire, USA). Readings were conducted by stacking the selected four leaves and positioning the stack within the leaf clip (model A122325, Malvern Panalytical Ltd., formerly Analytical Spectral Devices, Boulder, USA) of the instrument, which was connected to a plant probe (model A122317, Malvern Panalytical Ltd., formerly Analytical Spectral Devices, Boulder, USA). Stacking leaves in controlled measurements is a well-established experimental proxy for simulating dense canopy conditions and approaching the so-called infinite reflectance (R∞) - a condition where further increases in Leaf Area Index (LAI) no longer significantly alter leaf reflectance [69, 86]. This approach has been used to capture more ecologically realistic spectral responses from vegetation, particularly in the near-infrared region, and to minimize intraspecific variability. While this method does not account for the full three-dimensional structure or light-scattering dynamics of real canopies, it enables rapid and consistent measurements that balance ecological relevance with experimental feasibility. Therefore, we used stacked leaf reflectance (hereafter referred to as “leaf reflectance”) measurements to provide a practical approximation of canopy-level spectral behavior.

**Fig. 1:**
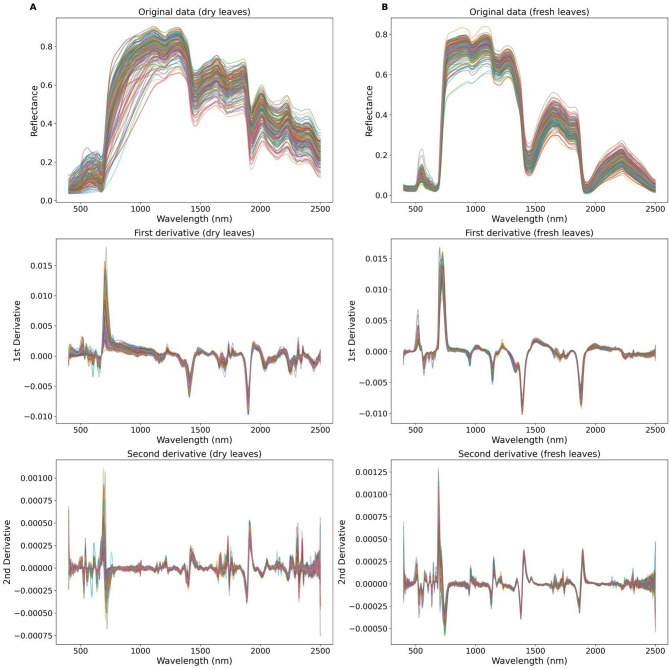
Raw hyperspectral reflectance data and first and second derivatives for (**A**) dry and (**B**) fresh leaves. A Savitzky-Golay filter was applied using a window length of nine for smoothing the raw data and a window length of 11 for the first and second derivatives, with a polynomial order of three. The different colors represent the average reading of each individual tree measured

A single reading on the adaxial side of the top leaf was recorded under an artificial light source, avoiding large primary or secondary veins but allowing smaller veins to be incorporated. After each reading, the top leaf was moved to the bottom of the stack, and the process was repeated, in a different part of the leaf lamina, until readings for all four leaves were obtained. The recorded spectra were then averaged to represent the spectral reading from that tree. Finally, these leaves were dried in an oven for 72 h at 60 ºC, and the same procedure was performed on the dry leaves. Spectral data discontinuities caused by changes between equipment sensors at 1000 nm and 1800 nm were corrected using the *jump_correct* function from the *specdal* library of Python 3 with Jupyter Notebook as the primary environment. Reflectance data at wavelengths 350–399 nm exhibited high noise levels and were removed and excluded from statistical analysis. Following that, hyperspectral data for dry and fresh leaves was smoothed by applying a Savitzky-Golay filter and calculating the first and second derivatives (Fig. 1) using a window length of nine for smoothing the raw data and a window length of 11 for the first and second derivatives, with a polynomial order of three.

### Statistical analysis

To isolate informative spectral bands while controlling for multicollinearity, we carried out stepwise feature selection in a Linear Discriminant Analysis (LDA) framework separately for each binary explanatory variable - presence/absence of isoprene *E*_c_, monoterpene storage, and sesquiterpene storage. For every variable, the procedure iteratively removed highly correlated neighboring bands and retained only those wavelengths that maximized the two‑class separation achieved by the LDA; the result was three non‑overlapping stepwise selected wavelength subsets, one per explanatory variable. In parallel, we applied derivative-based preprocessing (Savitzky-Golay filter) to our raw hyperspectral data to suppress low‑frequency background drift and attenuate high‑frequency noise created by collinear adjacent bands [53].

Following that, we fitted a total of 24 LDA models and 18 Random Forest (RF) models, representing the full factorial of (i) leaf‑condition datasets: dry or fresh leaf reflectance (2); (ii) binary explanatory variables: isoprene *E*_c_, monoterpene storage, or sesquiterpene storage (3); and (iii) spectral data inputs: LDA - raw data, first derivative, second derivative, or the stepwise selected subset (4)/ RF - raw data, first derivative, or second derivative (3). Each model, therefore, used one choice from each category (2 * 3 * 4 = 24 for LDA; 2 * 3 * 3 = 18 for RF).

Each individual model was trained and validated using non-overlapping subsets of the spectral data input: two-thirds of the observations (individual trees) were used for model training (training data), and the remaining one-third for validation (test data). To address imbalances in the number of observations between isoprene emitters and non-emitters, or mono-/sesquiterpene storing and non-storing trees, we applied the Random Under-Sampling (RUS) technique to the training data. This approach involves randomly removing observations from the majority class to create a balanced class distribution during model training. The test data were kept separate and untouched during this process to ensure an unbiased evaluation of model performance.

To examine the individual contribution of every wavelength chosen by the stepwise feature selection, we fitted univariate logistic regressions in which reflectance at a single wavelength was the predictor and the corresponding binary explanatory variable was the response. Significant results obtained in the logistic regressions were followed up with one‑way Kruskal-Wallis tests (non-parametric analysis of variance, ANOVA) to compare median reflectance values between isoprene emitters and non‑emitters, and mono-/sesquiterpene storing and non‑storing trees. This two‑step confirmatory analysis pinpoints the spectral regions that most strongly support class separation after accounting for multicollinearity.

Stepwise LDA selections were performed using R version 4.3.2 with RStudio as the primary environment. All other statistical analyses were performed in Python 3 with Jupyter Notebook as the primary environment. The Python libraries *pandas*, *NumPy*, *SciPy*, and *scikit-learn* were used for data handling and resampling, model construction, data splitting, and cross-validation; *statsmodels* for logistic regression models; and *matplotlib* and *seaborn* for visualizing results.

## Results

**Fig. 2:**
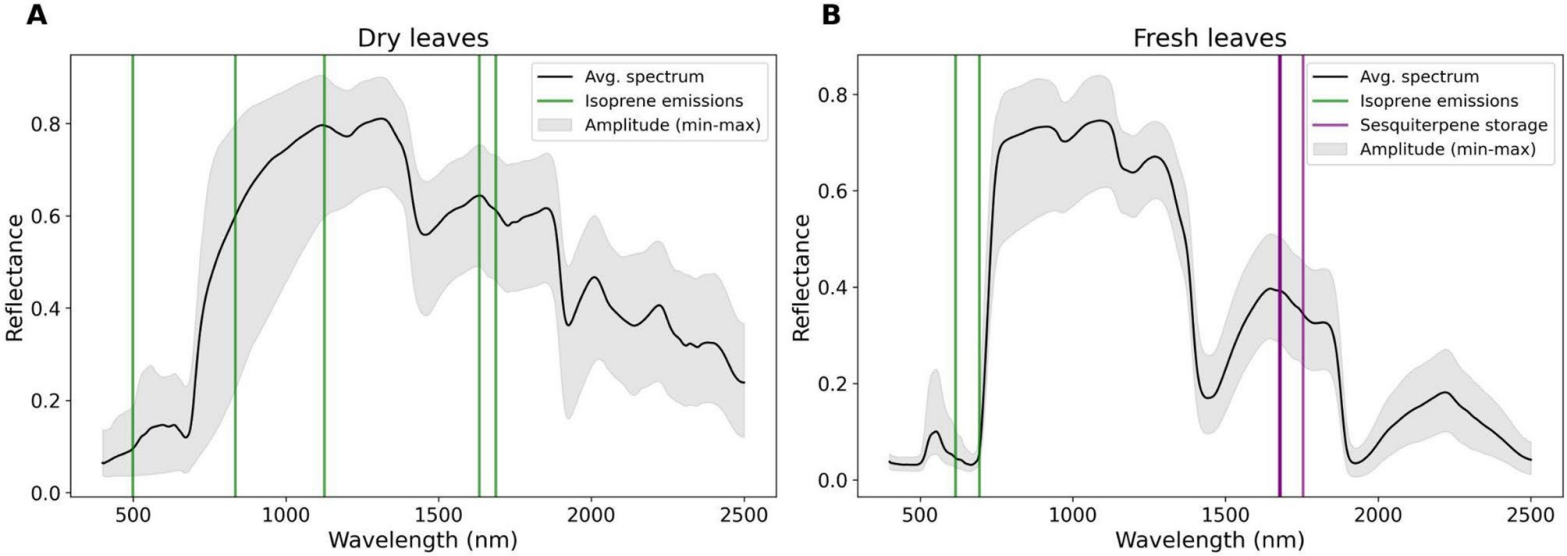
Average hyperspectral leaf reflectance spectrum (Avg. spectrum) obtained for (**A**) dry and (**B**) fresh leaves. Bars indicate wavelengths that were selected as most informative and also significantly contributed to distinguishing isoprene emitters and non-emitters (green) and sesquiterpene storing and non-storing trees (purple) in the logistic regression models

Wavelengths selected via stepwise LDA as most informative to predict isoprene emissions and mono-/sesquiterpene storage are presented in Table 2. Results from logistic regression models showed that, despite overlapping distributions, reflectance values in almost all wavelengths selected as most informative for isoprene emissions in dry and fresh leaves significantly contributed to distinguishing isoprene emitters from non-emitters. Meanwhile, only reflectance values from fresh leaves measured at wavelengths located in the short-wave region of the electromagnetic spectrum significantly distinguished sesquiterpene storing from non-storing trees in these models. None of the wavelengths selected as most informative for stored sesquiterpenes in dry leaves, and for stored monoterpenes in dry and fresh leaves, significantly differed between storing and non-storing trees (Table 2). Figure 2 represents the selected wavelengths that also significantly contributed to distinguishing isoprene emitters from non-emitters and sesquiterpene storing from non-storing trees in the logistic regression models.

**Table 2:** Wavelengths selected as most informative via stepwise Least Discriminant Analysis (LDA) selection for predicting isoprene emissions, and mono-/sesquiterpene storage for dry and fresh leaves. Region: the respective region they occupy in the electromagnetic spectrum; *p* value: *p* value of the logistic regression between reflectance values of isoprene emitters and non-emitters, and mono-/sesquiterpene storing and non-storing trees at the respective wavelengths; Sig.: statistical significance of the logistic regression at * *p* < 0.05 and ** *p* < 0.01; slope: slope of the logistic regression

		Wavelength	Region	*p* value	Sig.	Slope
***Isoprene emissions***	Dry	499	Visible	0.002	**	14.4
		835	Near-infrared	0.007	**	3.9
		1126	Near-infrared	0.001	**	9.6
		1633	Short-wave infrared	0.032	*	6.1
		1688	Short-wave infrared	0.023	*	6.7
		2499	Short-wave infrared	0.417		2.5
	Fresh	616	Visible	0.034	*	-28.1
		694	Visible	0.038	*	-25.2
		1155	Short-wave infrared	0.115		8.0
***Monoterpene storage***	Dry	716	Visible	0.402		-1.6
		969	Near-infrared	0.122		-2.7
	Fresh	766	Near-infrared	0.384		-2.5
		773	Near-infrared	0.389		-2.4
		1161	Near-infrared	0.320		5.0
		2175	Short-wave infrared	0.870		-0.7
		2177	Short-wave infrared	0.876		-0.7
***Sesquiterpene storage***	Dry	401	Visible	0.742		-3.1
		402	Visible	0.773		-2.7
		403	Visible	0.785		-2.5
		404	Visible	0.803		-2.3
		405	Visible	0.822		-2.0
		409	Visible	0.893		-1.2
		410	Visible	0.919		-0.9
		411	Visible	0.921		-0.8
		418	Visible	0.922		0.8
		434	Visible	0.667		2.9
		438	Visible	0.628		3.2
		444	Visible	0.589		3.4
		524	Visible	0.649		1.8
		528	Visible	0.644		1.8
		699	Visible	0.830		0.6
		749	Visible	0.947		0.1
		1429	Short-wave infrared	0.381		-2.6
		1894	Short-wave infrared	0.394		-2.4
	Fresh	400	Visible	0.564		-16.0
		401	Visible	0.518		-18.1
		406	Visible	0.606		-14.8
		408	Visible	0.663		-12.6
		409	Visible	0.619		-14.4
		427	Visible	0.807		-7.2
		432	Visible	0.790		-7.9
		437	Visible	0.800		-7.5
		668	Visible	0.904		-3.4
		678	Visible	0.785		-7.7
		747	Visible	0.490		-2.2
		1676	Short-wave infrared	0.036	*	-8.9
		1678	Short-wave infrared	0.037	*	-8.9
		1679	Short-wave infrared	0.037	*	-8.9
		1680	Short-wave infrared	0.037	*	-8.9
		1754	Short-wave infrared	0.042	*	-8.9

**Fig. 3:**
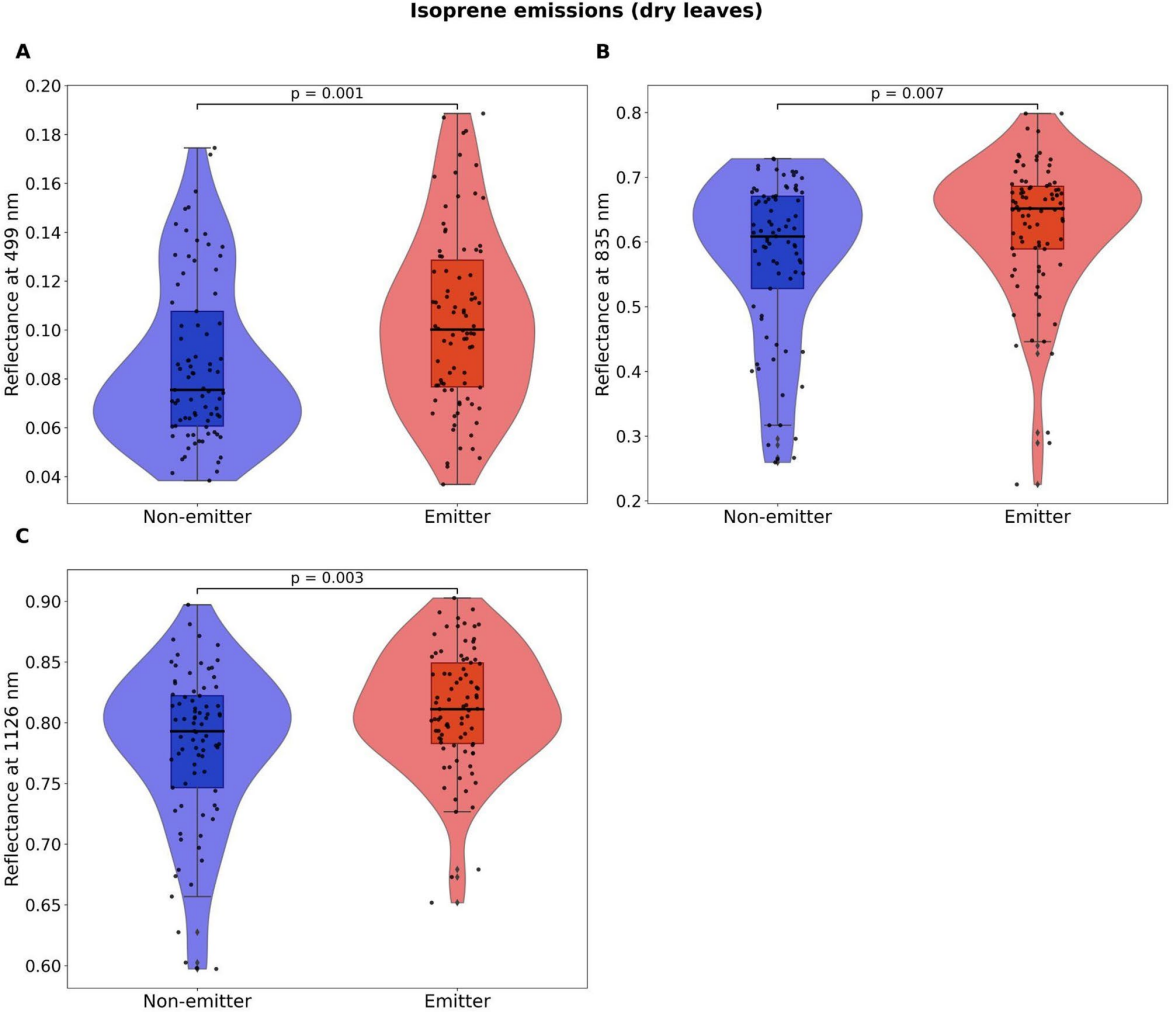
Results from Kruskal-Wallis tests (*n* = 167 trees) of dry leaf reflectance values at wavelengths that were selected as most informative via stepwise Least Discriminant Analysis (LDA) and significantly contributed to distinguishing isoprene emitters from non-emitters in the logistic regression models. Violin plots show the distribution and density of the observed data points, boxplots show the median and 25th and 75th percentiles, whiskers show the maximum and minimum acquired data points that were not considered outliers, and black circles represent the observed data points

**Fig. 4:**
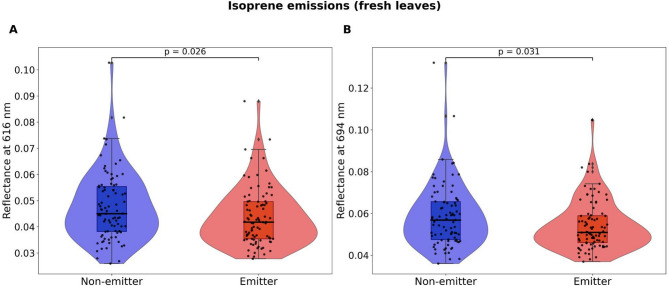
Results from Kruskal-Wallis tests (*n* = 175 trees) of fresh leaf reflectance values at wavelengths that were selected as most informative via stepwise Least Discriminant Analysis (LDA) and significantly contributed to distinguishing isoprene emitters from non-emitters in the logistic regression models. Violin plots show the distribution and density of the observed data points, boxplots show the median and 25th and 75th percentiles, whiskers show the maximum and minimum acquired data points that were not considered outliers, and black circles represent the observed data points

**Fig. 5:**
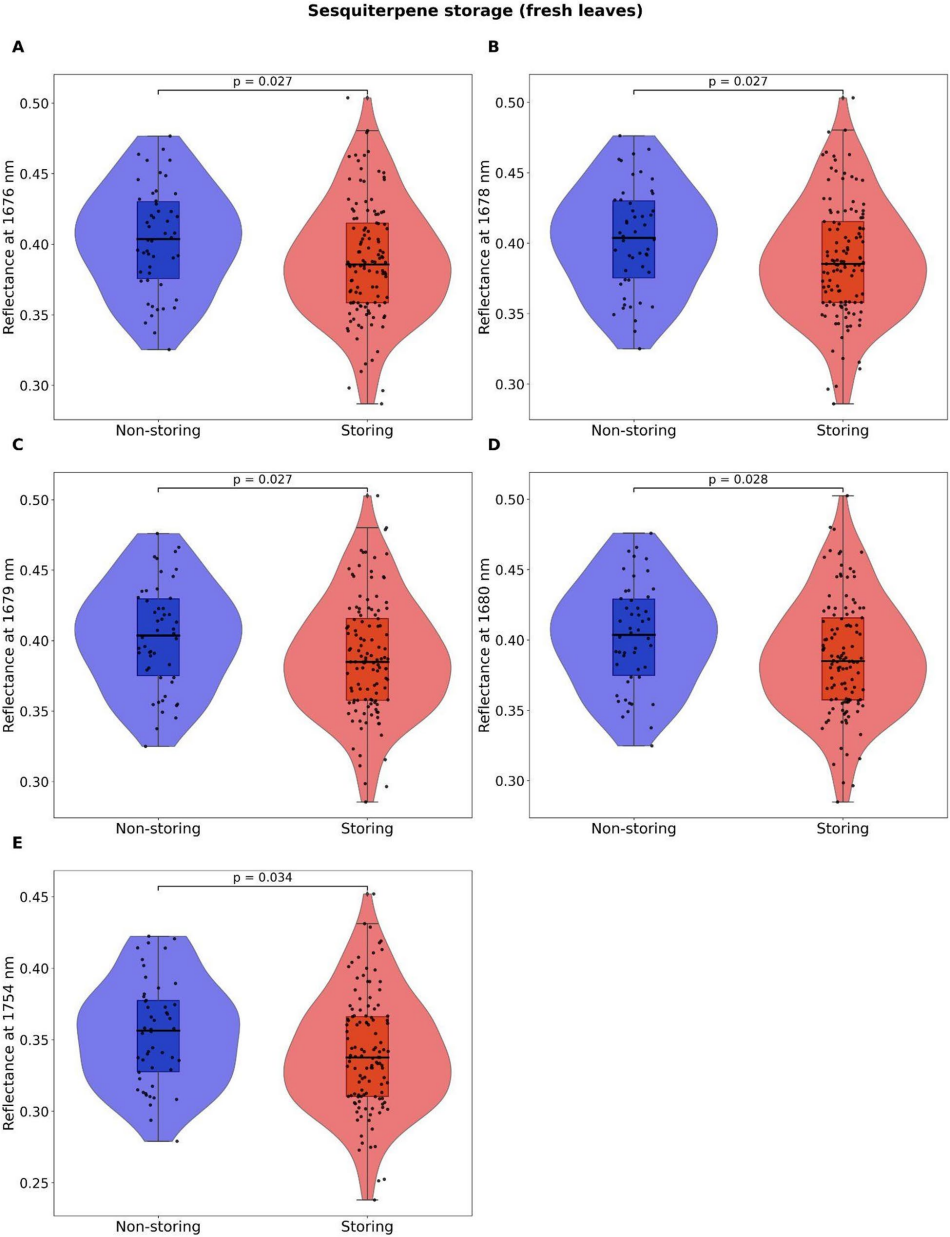
Results from Kruskal-Wallis tests (*n* = 171 trees) of fresh leaf reflectance values at wavelengths there were selected as most informative via stepwise Least Discriminant Analysis (LDA) and significantly contributed to distinguishing sesquiterpene storing from non-storing trees in the logistic regression models. Violin plots show the distribution and density of the observed data points, boxplots show the median and 25th and 75th percentiles, whiskers show the maximum and minimum acquired data points that were not considered outliers, and black circles represent the observed data points

Following logistic regression models, results from Kruskal-Wallis tests showed that, in isoprene emitters, dry leaves had significantly higher mean reflectance values at 499 (*p* = 0.001), 835 (*p* = 0.007), and 1126 (*p* = 0.003) (Fig. 3), while fresh leaves had lower mean reflectance values at 616 (*p* = 0.026) and 694 nm (*p* = 0.031) (Fig. 4). At the same time, fresh leaves from sesquiterpene-storing trees had significantly lower mean reflectance than non-storing trees at 1676 (*p* = 0.027), 1678-9 (*p* = 0.027), 1680 (*p* = 0.028) and 1754 nm (*p* = 0.034) (Fig. 5).

The results from LDA and RF discriminant models are displayed in Table 3. Discriminant models reached accuracies/F1-scores ranging from 0.47–0.72/0.44–0.75 for isoprene emissions, 0.47–0.65/0.41–0.65 for stored monoterpenes, and 0.35–0.67/0.27–0.78 for stored sesquiterpenes. LDA models tended to better separate isoprene emitters from non-emitters using dry leaf reflectance data, while RF models gave better results for fresh leaves. Still, the best model to predict isoprene emissions in fresh leaves was the LDA model performed with stepwise selected wavelengths (accuracy = 0.72, F1-score = 0.67, non-emitters / 0.75, emitters). LDA models performed with raw data and first derivative predicted isoprene emissions in dry leaves with an accuracy of 0.67 and F1-scores of 0.69 (non-emitters) / 0.64 (emitters), but first derivative models showed better F1-scores for isoprene emitters, meaning that they identified more true positives and fewer false negatives and had a better balance between precision and recall. Stored monoterpenes and sesquiterpenes were both better predicted from fresh leaf reflectance. Stored monoterpenes showed the highest accuracies (0.65) in the stepwise selection LDA and first derivative RF models, with the latter showing better recall (0.68) and F1-scores (0.65) for monoterpene storing trees. Lastly, the best model to predict the presence of sesquiterpene storage was the second derivative LDA model, with an accuracy of 0.67 and an F1-score of 0.78 for sesquiterpene storing-trees.

**Table 3:** Results from Least Discriminant Analysis (LDA) and Random Forest (RF) models using reflectance data from dry and fresh leaves to predict isoprene emissions, and monoterpene and sesquiterpene storage. LDA and RF models were constructed with combinations of (i) one leaf‑condition dataset: dry or fresh leaf reflectance; (ii) one binary explanatory variable: isoprene *E*_c_, monoterpene storage, or sesquiterpene storage; and (iii) one spectral data input: Raw data, first derivative, second derivative, or Stepwise selected wavelengths (LDA models)/ Raw data, first derivative, or second derivative (RF models). Precision: proportion of correctly identified positives out of all predicted positives; recall: proportion of correctly identified positives out of all actual positives; F1-score: balance between precision and recall, calculated as 2 * (precision * recall/ precision + recall) [87]. Underlined values indicate models with the highest accuracies and F1-scores

*Isoprene emissions*			Raw hyperspectral data			1st Derivative			2nd Derivative			Stepwise selection		
			Precision	Recall	F1-score	Precision	Recall	F1-score	Precision	Recall	F1-score	Precision	Recall	F1-score
LDA	Dry	Non-emitter	0.72	0.70	0.71	0.76	0.63	0.69	0.70	0.63	0.67	0.75	0.60	0.67
		Emitter	0.59	0.62	0.60	0.58	0.71	0.64	0.54	0.62	0.58	0.56	0.71	0.63
		**Accuracy**			**0.67**			**0.67**			**0.63**			**0.65**
	Fresh	Non-emitter	0.50	0.55	0.52	0.39	0.50	0.44	0.43	0.41	0.42	0.65	0.68	0.67
		Emitter	0.66	0.61	0.63	0.56	0.45	0.50	0.59	0.61	0.60	0.77	0.74	0.75
		**Accuracy**			**0.58**			**0.47**			**0.53**			**0.72**
RF	Dry	Non-emitter	0.65	0.57	0.61	0.69	0.60	0.64	0.72	0.60	0.65			
		Emitter	0.48	0.57	0.52	0.52	0.62	0.57	0.54	0.67	0.60			
		**Accuracy**			**0.57**			**0.61**			**0.63**			
	Fresh	Non-emitter	0.56	0.82	0.67	0.46	0.50	0.48	0.56	0.68	0.61			
		Emitter	0.81	0.55	0.65	0.62	0.58	0.60	0.73	0.61	0.67			
		**Accuracy**			**0.66**			**0.55**			**0.64**			
***Monoterpene storage***														
LDA	Dry	Non-storing	0.71	0.50	0.59	0.74	0.57	0.64	0.59	0.53	0.56	0.65	0.73	0.69
		Storing	0.46	0.68	0.55	0.50	0.68	0.58	0.36	0.42	0.39	0.47	0.37	0.41
		**Accuracy**			**0.57**			**0.61**			**0.49**			**0.59**
	Fresh	Non-storing	0.67	0.59	0.63	0.60	0.56	0.58	0.57	0.63	0.60	0.66	0.70	0.68
		Storing	0.61	0.68	0.64	0.56	0.60	0.58	0.55	0.48	0.51	0.65	0.60	0.63
		**Accuracy**			**0.63**			**0.58**			**0.56**			**0.65**
RF	Dry	Non-storing	0.58	0.47	0.52	0.67	0.60	0.63	0.72	0.60	0.65			
		Storing	0.36	0.47	0.41	0.45	0.53	0.49	0.50	0.63	0.56			
		**Accuracy**			**0.47**			**0.57**			**0.61**			
	Fresh	Non-storing	0.63	0.44	0.52	0.68	0.63	0.65	0.58	0.67	0.62			
		Storing	0.55	0.72	0.62	0.63	0.68	0.65	0.57	0.48	0.52			
		**Accuracy**			**0.58**			**0.65**			**0.58**			
***Sesquiterpene storage***														
LDA	Dry	Non-storing	0.30	0.38	0.33	0.38	0.38	0.38	0.38	0.56	0.45	0.21	0.38	0.27
		Storing	0.66	0.58	0.61	0.70	0.70	0.70	0.72	0.55	0.62	0.52	0.33	0.41
		**Accuracy**			**0.51**			**0.59**			**0.55**			**0.35**
	Fresh	Non-storing	0.22	0.50	0.30	0.18	0.30	0.22	0.27	0.40	0.32	0.21	0.50	0.29
		Storing	0.83	0.57	0.68	0.80	0.67	0.73	0.84	0.74	0.78	0.82	0.55	0.66
		**Accuracy**			**0.56**			**0.60**			**0.67**			**0.54**
RF	Dry	Non-storing	0.29	0.25	0.27	0.27	0.25	0.26	0.26	0.31	0.29			
		Storing	0.66	0.70	0.68	0.65	0.67	0.66	0.63	0.58	0.60			
		**Accuracy**			**0.55**			**0.53**			**0.49**			
	Fresh	Non-storing	0.14	0.30	0.19	0.14	0.30	0.19	0.14	0.30	0.19			
		Storing	0.77	0.55	0.64	0.77	0.57	0.66	0.77	0.57	0.66			
		**Accuracy**			**0.50**			**0.52**			**0.52**			

## Discussion

The results from our study showed that hyperspectral visible to short-wave infrared (VSWIR) reflectance is a useful tool to predict the presence of isoprene emissions and terpene storage from leaves of central Amazon Forest trees. Least Discriminant Analysis (LDA) models built with first derivative dry leaf reflectance showed the capacity to predict the presence of isoprene emissions with an accuracy of 0.67 and F1-scores of 0.69 (non-emitters) and 0.64 (emitters). These results suggest the use of spectral readings from herbarium specimens to assist in the development of more efficient sampling designs targeted at potential isoprene emitters. Still, LDA models built with fresh leaf reflectance measured at specific wavelengths (616, 694, and 1155 nm) - selected as most informative through stepwise feature selection - reached even higher accuracy (0.72) and F1-scores (0.67, non-emitters; 0.75, emitters), which suggests that key spectral features (most informative wavelengths) could be potentially further incorporated into remote sensing models to detect potential isoprene-emitting trees.

Although logistic regression models with wavelengths selected as most informative to predict the presence of monoterpene storage were not statistically significant, Random Forest (RF) models using first derivative fresh leaf reflectance data predicted the presence of monoterpene storage with an accuracy of 0.65 and F1-scores of 0.65 for both non-storing and storing trees. Finally, mean reflectance values obtained at 1676, 1678-80, and 1754 nm significantly differed between sesquiterpene-storing and non-storing trees. However, unlike isoprene, the highest accuracy (0.67) and F1-score (0.78) in sesquiterpene storage discriminant models were observed in LDA models constructed with second derivative fresh leaf reflectance. Results observed for mono-/sesquiterpene storage discriminant models indicate the potential use of fresh leaf reflectance spectroscopy as a preprocessing tool to orient sampling efforts in the field toward potential terpene-storing trees.

### Specific wavelength reflectance differences in isoprene emitters and sesquiterpene-storing trees

Wavelengths that were selected as most informative for the presence of monoterpene storage did not significantly contribute to distinguishing monoterpene storing from non-storing trees. Therefore, we centered the following discussion around wavelengths that were selected as most informative in stepwise feature LDAs and also significantly distinguished isoprene emitters from non-emitters, and sesquiterpene storing from non-storing trees in the logistic regression models. These models showed that isoprene emitters and non-emitters can be significantly distinguished using dry leaf reflectance measured at wavelengths related to photosynthetic pigments (carotenoids, 499 nm; chlorophyll, 835 nm) [69, 88–90], water content (1126 nm) [90–92], and cellulose and lignin contents (1633 and 1688 nm) [69, 90]. Associations between the presence of isoprene emission and increases in leaf dry matter and accumulation of lignin have been previously demonstrated [6, 74, 75], which corroborates these relationships between the presence of isoprene emissions and water, cellulose, and lignin content-related wavelengths. Meanwhile, Kruskal-Wallis tests revealed non-statistically significant differences in mean dry leaf reflectance values between isoprene emitters and non-emitters at cellulose and lignin-related wavelengths. However, dry leaves from isoprene emitters showed significantly higher reflectance at wavelengths related to carotenoids and chlorophyll [69, 88–90].

Carotenoids are produced in the same biochemical pathway as isoprene, meaning that their production also depends mostly on the supply of photosynthetic carbon upstream of the chloroplastic MEP pathway [33–36, 93]. Considering this, isoprene-emitting leaves would be expected to show higher concentrations of chlorophyll and carotenoids, and consequently higher absorption (lower reflectance) at these wavelengths. In fact, our results showed that fresh isoprene-emitting leaves reflected less (and possibly absorbed more) in chlorophyll absorption-related wavelengths (616 and 694 nm) [69]. Even though we did not directly measure transmittance in our study, stacked leaf measurements simulate high-LAI canopies, which minimize light transmission [69, 86]. Hence, the reduced reflectance values we observed were likely linked to increased light absorption. However, dry leaves from isoprene emitters reflected more (or absorbed less) at these wavelengths, and we suggest that this might be due to artifacts from the leaf desiccation process. It is possible that leaf desiccation caused alterations in tissue structure and pigment composition differently in isoprene emitters, as it can promote the collapse of internal leaf structures or the degradation of pigments in ways that enhance light scattering, as well as promote the production of residual isoprenoid compounds that may increase reflectance at these wavelengths [94, 95]. Therefore, we propose that more detailed studies are needed to better elucidate the relationships between isoprene emissions and reflectance from dry leaves at these specific wavelengths.

Finally, fresh leaves from sesquiterpene-storing trees reflected significantly less (or absorbed more) at wavelengths related to phenolic compounds (1676, 1678-80, and 1754 nm) [65, 90, 96]. This suggests that the presence of sesquiterpene storage in these leaves might be associated with a higher phenolics content and overall higher investments in functionally diverse, chemical-based defenses. Indeed, sesquiterpene-storing trees showed a marginally significant tendency of higher total phenolics content compared to non-storing trees (*p* = 0.085; Fig. S1). Both phenolic and sesquiterpene compounds are highly involved in herbivore defense, with the latter playing active signaling roles in plant communication as well [12–15], and such an association may be very useful in such a species-rich, ecologically complex forest like the Amazon.

### Leaf reflectance spectroscopy as a tool to predict the presence of isoprene emissions and terpene storage

The fact that isoprene is not a storable compound is probably an important factor preventing our discriminant models from reaching very high accuracies. Still, our results demonstrated that the presence of isoprene emissions in leaves from central Amazon Forest trees can be predicted by first derivative dry leaf reflectance (accuracy = 0.67; F1-score = 0.69, non-emitters / 0.64, emitter), and fresh leaf reflectance measured at only three wavelengths: 616, 694, and 1155 nm (accuracy = 0.72; F1-score = 0.67, non-emitters / 0.75, emitters). We suggest that reflectance spectroscopy likely indirectly predicted the presence of isoprene emissions in dry leaves due to its strong positive relationship with leaf structural traits such as dry matter and lignin contents [6, 74, 75]. Similarly, the high predictive power of the fresh leaf stepwise selected-reflectance model was likely due to the intrinsic dependence of isoprene production on photosynthesis [33–36] - as higher carbon assimilation rates translate into higher emission rates in isoprene-emitting trees [33, 34] - and to isoprene’s relationship with chlorophyll and carotenoid contents [95], as both can be directly assessed by reflectance spectroscopy [88, 89, 97].

Our discriminant models showed that the presence of mono-/sesquiterpene storage can also be predicted - with accuracies of 0.65 (F1-score = 0.65 for non-storing and storing) and 0.67 (F1-score = 0.32, non-storing / 0.78, storing), respectively - by fresh leaf reflectance measurements. Dry leaf reflectance models possibly showed comparable lower predictive capacities due to the leaf desiccation process, which tends to promote the loss of some of the more volatile mono-/sesquiterpene compounds that are stored within the plant cell wall and encounter less diffuse resistance [98]. Also, even though we applied an undersampling function to all models in order to balance the number of observations in each class, the outputs from sesquiterpene storage models were still considerably skewed toward sesquiterpene-storing trees (F1-score = 0.32, non-storing / 0.78, storing), which were much more prevalent in our dataset.

At the same time, the categories of mono-/sesquiterpene storage contained, respectively, 14 and 25 different identified compounds (Table S1), but logistical difficulties in obtaining appropriate calibration standards for the chemical analysis of these compounds [75] prevented the quantification of the magnitude of each compound. Considering that trees contained diverse mixes of mono-/sesquiterpene compounds with varying molecular structures [99] and that sesquiterpene storage is widespread in central Amazon Forest trees [75], we suggest that leaf reflectance spectroscopy may be better suited to estimate the concentration of specific compounds rather than detect binary presence/absence. This is supported by the continuous nature of reflectance spectra, which can capture subtle, nonlinear variations linked to compound concentration [100]. Furthermore, we propose that future research should focus more deeply on the development of reflectance-based models to predict concentrations of different mono-/sesquiterpene compounds, since these are highly emitted under stressed conditions [32, 101], contribute to higher particle formation than isoprene [20], and different compounds have distinct effects on atmospheric processes (e.g., trans-β-ocimene is a monoterpene that yields higher O_3_ formation in polluted atmospheres [32]).

Another factor to take into account is that, ultimately, the strongest determinants of the presence of VI emissions and storage are isoprene (*IspS*) and terpene synthase (*TPS-b*) encoding genes - which are conserved at the taxonomic species level; however, while *TPS-b* genes are widespread in plant lineages, *IspS* genes do not show a clear phylogenetic thread [84, 85]. Even though reflectance spectroscopy can successfully discriminate taxonomic species [52–55], its effectiveness in our study may have been limited by a few factors. These include the great diversity of species we measured, the very limited number of replicates per species available in our sampling plot, and possible taxonomic uncertainties - which are quite common given the many non-monophyletic groups and cryptic species in the Amazon Forest [42]. These challenges likely limited the capacity of our discriminant models to reach exceptionally high accuracies (> 0.9).

Moreover, while we focused on adaxial leaf surface measurements - as these are better suited for remote sensing models [60, 65, 66, 70, 71, 96, 102] - studies have observed that a combination of adaxial and abaxial reflectance measurements worked better at discriminating taxonomic species [53, 54]. They argue that, because of epidermal differences between leaf surfaces, more morphological information can be added to species identification models when inputting spectral data from both surfaces - even though they also achieved > 99% mean correct identifications with only adaxial surface measurements [53]. Considering this and the phylogenetic aspect of VI emissions, we propose that future studies incorporating both adaxial and abaxial reflectance measurements, and a sampling design with enough species repetition and well-verified taxonomic identifications, could enhance the ability of spectral models to simultaneously retrieve taxonomic species information, thereby further improving predictions of VI emissions and storage.

Nevertheless, our study demonstrated that leaf reflectance spectroscopy predicted the presence of isoprene emissions and mono-/sesquiterpene storage with accuracies and F1 scores equal to or higher than 0.64. These findings indicate the possibility of, for instance, obtaining spectral readings from botanical collections or field inventories to develop sampling designs targeted at potential isoprene-emitting or terpene-storing trees, or using key spectral features (most informative selected wavelengths) for potential future incorporation into remote sensing models to detect potential isoprene-emitters. Accurately measuring VI emissions at leaf, canopy, and ecosystem scales is a difficult, time-consuming task - particularly in remote and often inaccessible regions of the Amazon Forest. It involves very specific and expensive instrumentation (e.g., proton-transfer reaction mass spectrometry, chromatography-mass spectrometry) as well as the establishment of large-scale tower systems for canopy and ecosystem flux monitoring, or the use of canopy cranes and the hard work of tree climbers for leaf-level measurements. Moreover, satellite retrievals (i.e., isoprene) and VI emission model estimates are prone to high levels of uncertainty given the lack of observational studies to parameterize and validate models. Therefore, the use of simple, less time-consuming, spectral tools for predicting potential VI emitters could be extremely helpful in increasing available VI emission data, reducing modeling emission uncertainties, and contributing to understanding the roles of VIs within forest-atmosphere interactions, atmospheric chemistry, and the carbon cycle.

## Conclusions

In this study, we demonstrated that leaf reflectance spectroscopy is a useful tool to predict the presence of isoprene emissions and terpene storage across a broad selection of Amazonian tree species. By either guiding sampling efforts based on reflectance-based discriminant models or identifying key spectral features for a potential future incorporation into remote sensing models, our results show that leaf reflectance spectroscopy may provide a straightforward, scalable approach for identifying potential VI emitters, particularly in remote tropical forests like the Amazon. Such an approach may translate into more refined VI emission estimates, lower modeling uncertainties, and a deeper understanding of the crucial roles that these compounds play in carbon cycling, atmospheric chemistry, and climate regulation.

## Electronic supplementary material

Below is the link to the electronic supplementary material.

Supplementary Material 1

## Data Availability

The isoprene emission capacity and terpene storage data analyzed in this study can be found in: 10.17871/atto.363.7.1695; and the hyperspectral leaf reflectance data generated and analyzed for this study is available at the ATTO Data Portal in: https://www.attodata.org/ddm/data/Showdata/458.
