# Supplementary material for: Leaf spectroscopy as a tool for predicting the presence of isoprene emissions and terpene storage in central Amazon forest trees
Source: Plant Methods. 2025 Jun 4;21:78. doi: 10.1186/s13007-025-01400-w (PMC12135534; doi:10.1186/s13007-025-01400-w)
Supplement: Supplementary file 1 — Supplementary Material 1 [file 13007_2025_1400_MOESM1_ESM.docx]

**Additional supporting information for**

**Leaf spectroscopy as a tool for predicting the presence of isoprene emissions and terpene storage in central Amazon forest trees**

**Michelle Robin^1*^, Flavia Machado Durgante^2,4^, Caroline Lorenci Mallmann^3^, Hilana Louise Hadlich^4^, Christine Römermann^5,6,7^, Lucas de Souza Falcão^8^, Caroline Dutra Lacerda^8^, Sérgio Duvoisin Júnior^8^, Florian Wittmann^2^, Maria Teresa Fernandez Piedade^4^, Jochen Schöngart^4^, Eliane Gomes Alves^1,9*^**

^1^Biogeochemical Processes Department, Max Planck Institute for Biogeochemistry, Jena, Germany

^2^Department of Wetlands Ecology, Karlsruhe Institute of Technology, Karlsruhe, Germany

^3^Department of Geosciences, Federal University of Santa Maria, Santa Maria, Brazil

^4^Department of Botany, National Institute of Amazonian Research, Manaus, Brazil

^5^Institute for Ecology and Evolution, Friedrich-Schiller University, Jena, Germany

^6^German Centre for Integrative Biodiversity Research (iDiv) Halle-Jena-Leipzig, Germany

^7^Senckenberg Institute for Plant Form and Function (SIP), Jena, Germany

^8^Department of Chemistry, University of Amazonas State, Manaus, Brazil

^9^Department of Climate and Environment, National Institute of Amazonian Research, Manaus, Brazil

**corresponding authors:* [mcarneiro@bgc-jena.mpg.de](mailto:mcarneiro@bgc-jena.mpg.de), [egomes@bgc-jena.mpg.de](mailto:egomes@bgc-jena.mpg.de)


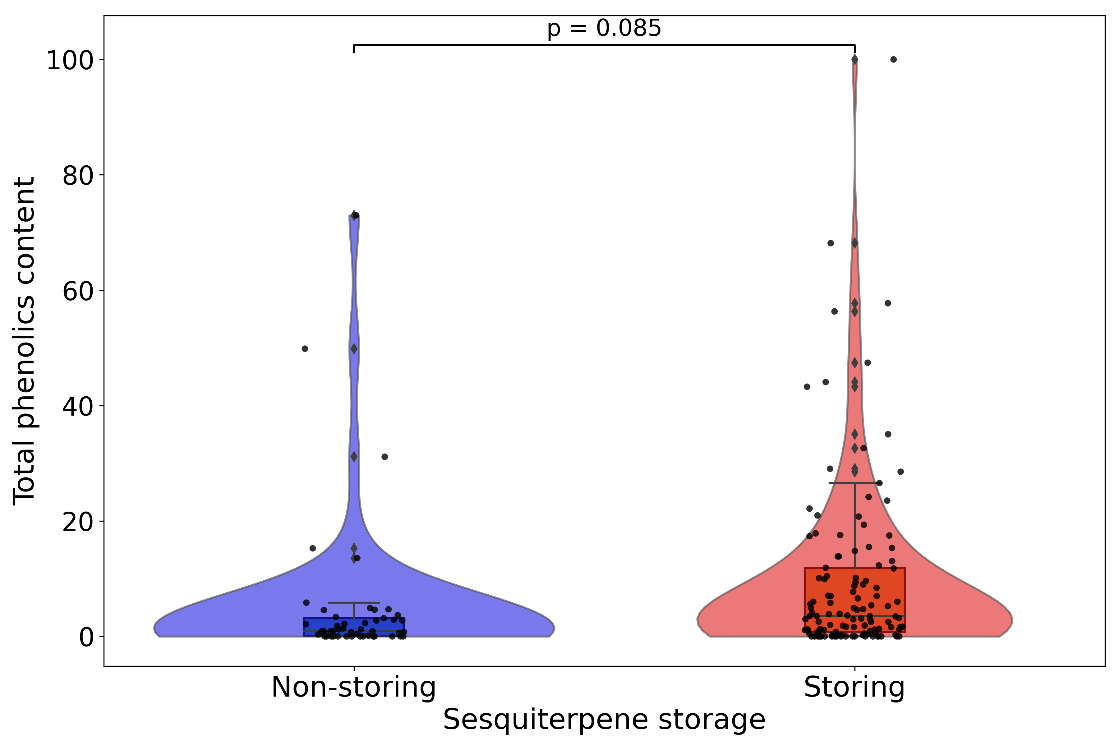


**Figure S1.** Results from logistic regression model (*n* = 169 trees) between total phenolics content and presence of sesquiterpene storage. Total phenolics measurements are described in Robin *et al*. (2024) and data can be found in: 10.17871/atto.363.7.1695. Violin plots show the distribution and density of the observed data points, boxplots show the median and 25th and 75th percentiles, whiskers show the maximum and minimum acquired data points that were not considered outliers, and black circles represent the observed data points.

**Table S1.** List of detected stored monoterpenes and sesquiterpenes and number (*n*) of trees in which each compound was detected, adapted from Robin *et al*. (2024).

| **Stored monoterpenes** | | **Stored sesquiterpenes** | |
| --- | --- | --- | --- |
| **Compound** | ***n* of trees** | **Compound** | ***n* of trees** |
| Limonene | 56 | Caryophyllene | 97 |
| Linalool | 39 | Copaene | 94 |
| p-Cymene | 35 | α-Calacorene | 31 |
| α-Terpineol | 33 | Alloaromadendrene | 31 |
| α-Pinene | 17 | α-Cubebene | 27 |
| γ-Terpinene | 16 | α-Muurolene | 24 |
| β-Ocimene | 13 | *cis*-α-Bergamotene | 23 |
| Terpinen-4-ol | 10 | Globulol | 22 |
| Camphene | 7 | γ-Muurolene | 18 |
| Eucalyptol | 7 | Ylangene | 17 |
| α-Phellandrene | 5 | Aromandendrene | 16 |
| *p*-Menthatriene | 5 | Selina-3-7-11-diene | 13 |
| β-Myrcene | 4 | α-Guaiene | 13 |
| endo-Borneol | 4 | α-Maaliene | 12 |
|  |  | τ-Muurolol | 11 |
|  |  | Guaiol | 11 |
|  |  | β-Bourbonene | 10 |
|  |  | τ-Cadinol | 8 |
|  |  | Humulene | 8 |
|  |  | Isoledene | 8 |
|  |  | γ-Elemene | 7 |
|  |  | *trans*-Calamenene | 7 |
|  |  | *cis*-Muurola-4-15-5-diene | 6 |
|  |  | Neointermedeol | 5 |
|  |  | β-Bisabolene | 4 |

**Method S1.** Description of isoprene emission capacity (*E*_c_) measurements.

Leaf-level isoprene *E*_c_ measurements were obtained in real-time under defined environmental conditions (photosynthetic photon flux density of 1000 μmol m^-2^ s^-1^, leaf temperature of 30 ºC, flow rate of air going into the leaf chamber of 400 μmol s^-1^, CO_2_ and H_2_O concentrations of 420 μmol mol^-1^ and 21 mmol mol^-1^ and relative humidity of ~60 %) using a combined system consisting of a LI-6800 portable gas exchange (LiCor Inc., USA) connected to proton-transfer-reaction quadrupole mass spectrometer (PTR-QMS, IONICON Analytik, Innsbruck, Austria) as described in Robin et al. (2024). A hydrocarbon filter (Restek Pure Chromatography, Restek Corporations, USA) was installed at the air inlet of the LI-6800 to remove isoprene from incoming ambient air. All tubing in contact with the sampling air was PTFE and did not exchange isoprene. At the beginning of each day and before each measurement, a chamber blank sample was obtained from the empty leaf chamber. Measurements were performed by separately enclosing one leaf (for compound leaves we considered a leaflet as the equivalent of a simple leaf lamina) in the LI-6800 leaf chamber under the standard environmental conditions defined above. The stability criterion for measurements was defined as one standard deviation of the mean *A*_n_ and *g*_s_, and these variables were visually monitored until the value reached a stable plateau (~ 10 min), with measurements beginning when the instrument had reached the defined stability criterion. If the stability criterion was not met, we replaced the leaf or sampled a new branch. The air exiting the sample port located behind the LI-6800 head was redirected to the PTR-QMS, which operated in standard conditions with a drift tube voltage of 600 V, drift tube pressure of 2.2 mbar, and E/N 120 Td. Measurements were performed for 10 minutes, and during each PTR-QMS measurement cycle the following mass-to-charge ratios (m/z) were monitored: 21 (H_3_^18^O_+_), 32 (O_2_^+^), and 37 (H_2_O-H_3_O^+^) with a dwell time of 500 ms each; 41 (isoprene fragment), 69 (isoprene) with a dwell time of 1 s each. Humidity-dependent calibrations (using water-bubbled nitrogen to dilute standard gas, simulating ambient relative humidity) were performed with a certified isoprene standard gas (Apel-Riemer Environmental, Inc.) at the beginning and end of the measurement campaign. The mixing ratios of isoprene were calculated from the calibration curves (R^2^ = 0.99). The detection limit of the PTR-QMS was calculated as three times the standard deviation of isoprene (ppb) detected in the water-bubbled nitrogen background of the calibration curves and was equal to 0.93 ppb. Once mixing ratios of isoprene (ppb) from the samples were obtained, isoprene emission capacity was determined using the equation (*E*_c_ = *Rppb* × Q/S), where *E*_c_ (nmol m^-2^ s^-1^) is the leaf flux of isoprene emission; *Rppb* (nmol mol^-1^) is isoprene concentration of the outgoing air; Q is the flow rate of air into the leaf chamber (400 x 10^-6^ mol s^-1^); S is the area of leaf within the chamber (0.0002 m² or 0.0006 m²).

**Method S2.** Description of stored monoterpene and sesquiterpene measurements.

Contents of monoterpenes and sesquiterpenes stored in leaves were obtained from ~2 g of fresh leaf material macerated in liquid nitrogen and transferred to a 20 mL glass vial as described in Robin et al. (2024). Samples were analyzed via gas chromatography with a headspace mass-spectrometry detector (Headspace/GC-MS) system at the Chemical Analysis Lab (State University of Amazonas - UEA). Before each injection, the automatic sampler (AOC-6000 plus, Shimadzu, Kyoto, Japan) transferred the glass vial to the headspace oven and the vial was heated to 150 °C for 20 min at 250 rpm. Following that, the gas phase of the sample was extracted with a hermetic needle at 150 ºC and injected into the column of a gas chromatography-mass spectrometer GC-MS-TQ8050 NX (Shimadzu, Kyoto, Japan). The injection was split (1:10), and the trap was heated to 200 ºC while backflushing with a carrier gas (helium) at a flow rate of 5.2 ml min^-1^ (linear speed of 37.4 cm s^-1^ and pressure of 53.5 kPa) directed into the column (SH-I-5Sil MS, 5% diphenyl / 95% dimethyl pol, 30.0 m length x 0.25 mm inner diameter x 0.25 μm film thickness). The oven ramp temperature was programmed with an initial hold of 5 min at 35 ºC, followed by an increase to 280 ºC at a rate of 5 ºC min^-1^ followed by a hold at 280 ºC for 36 min. The interface was at 290 °C and the ion source was at 200 °C. A Shimadzu gas quality workstation (GC-MS solution v. 4.53) and NIST20 Mass Spectral Library were used to analyze the chromatographic data. The peak integration parameters were set as follows: slope (S) was 100, peak width at half-height (W) was 3, drift (D) was 0, parameter change time (T) was 1000, minimum peak area (M) was 1000, smoothing (F) was 1, and smoothing peak width at half-height (O) was 1. With these peak integration parameters, chromatographic peaks were automatically integrated. The mass spectrum of each peak was compared with the standard spectrums in the NIST20 spectrum library, and compounds with a similarity score of more than 90 were selected. Values were calculated as percentages of relative abundance of stored monoterpenes and stored sesquiterpenes by summing the peak areas of stored monoterpenes (sum of stored monoterpenes) and stored sesquiterpenes (sum of stored sesquiterpenes) found in a given sample and normalizing each sum by the largest sum observed in the dataset for each group of compounds.
